# Occult hepatitis B infection in egyptian chronic hepatitis C patients: prevalence, impact on pegylated interferon/ribavirin therapy

**DOI:** 10.1186/1743-422X-7-324

**Published:** 2010-11-17

**Authors:** Mohamed H Emara, Nahla E El-Gammal, Lamiaa A Mohamed, Maged M Bahgat

**Affiliations:** 1 Tropical Medicine Department, Faculty of Medicine, Zagazig University, Zagazig, Egypt; 2 Clinical Pathology Department, Faculty of Medicine, Zagazig University, Zagazig, Egypt

## Abstract

**Background:**

Chronic HCV infection combined with occult hepatitis B infection has been associated with liver enzymes flare, advanced hepatic fibrosis and cirrhosis, poor response to standard interferon-α, and increased risk of HCC. This study aimed to elucidate the prevalence of occult hepatitis B infection in Egyptian chronic HCV patients, and to clarify its role in non-response of those patients to pegylated interferon/ribavirin therapy. This study enrolled 155 consecutive chronic HCV patients under pegylated interferon/ribavirin therapy. All patients were exposed to clinical assessment, biochemical, histological and virological examinations. HBV parameters (HBV DNA, anti-HBc, anti-HBs) and patients' response status to the combination therapy were determined.

**Results:**

In this study, occult hepatitis B infection occurs in 3.9% of Egyptian chronic HCV patients; tends to affect younger age patients, associated with higher base line HCV viral load, less hepatic fibrosis than monoinfected patients. This occult hepatitis B infection is not a statistically significant cause of non-response to pegylated interferon/ribavirin therapy. Anti-HBs was not associated with any biochemical, histological or virological abnormalities in those patients, contrary to low response rate to therapy and higher HCV viral load that was observed with anti-HBc.

**Conclusions:**

Detection of HBV DNA in HBsAg negative chronic HCV patients plays a non significant role in non-response of Egyptian patients to pegylated interferon/ribavirin therapy.

## Background

Chronic hepatitis C (HCV) affects more than 170 million people worldwide, causing cirrhosis and liver cancer [1]. In Egypt, high HCV rates were reported reaching up to 20% [2]. The currently recommended therapy for chronic HCV is the combination of pegylated interferon alpha and ribavirin (Peg-IFN/RBV) that proved to be superior to standard interferon alpha and ribavirin [3]. More than 50% of patients can achieve a sustained virological response (SVR) after 24-48 weeks of this combination therapy, making HCV a potentially curable disease [1]. For patients with HCV genotype 4 infections (the prevalent genotype in Egypt), combination treatment with pegylated interferon alpha and weight based ribavirin administered for 48 weeks seems to be an appropriate regimen [4]. Occult hepatitis B virus infection (OBI) is defined as the presence of HBV DNA, in serum and/or the liver tissue without detectable HBsAg with or without anti-HBc or anti-HBs outside the pre-seroconversion window period [5]. Both HBV and HCV share common routes of transmission and hence there is a consensus that patients with chronic HCV liver disease, are at high risk of OBI [6,7]. OBI may contribute to liver inflammation through episodes of increased viral replication, increased immune activity and subsequent liver injury [8].

In chronic HCV infection, the presence of OBI has been associated with liver enzymes flare [8], increased severity of liver disease towards advanced fibrosis and cirrhosis [6,9], poor response to standard interferon-α in many [6,9-12], but not all [13] studies, and increased risk of HCC [14,15].

Although the potential mechanism for reduced interferon response in these cases remains unclear, one intriguing investigation has shown decreased intrahepatic expression of interferon receptor mRNA and protein in OBI [12].

Some studies suggested a negative influence of OBI on the response to standard interferon in chronic HCV infection [6,9-12]. This observation needs to be confirmed in HCV populations treated with the standard of care Peg-IFN/RBV combination therapy [16].

This study aimed to elucidate the prevalence of OBI in Egyptian chronic HCV patients, and to clarify its role as a cause of non-response of those patients to the standard of care Peg-IFN/RBV therapy.

## Patients and methods

The ethical committee of our institution approved this study to be conducted at both Al-Ahrar General Hospital and Zagazig University Hospitals, Sharkia Governorate, Egypt, and included 155 chronic HCV patients under Peg-IFN/RBV therapy. The diagnosis of HCV was confirmed by detection of anti-HCV antibody and HCV RNA and they were all candidates to begin the combination therapy according to the guidelines of the national Committee for Control and Prevention of viral Hepatitis "C" in Egypt, with the following criteria:

### Inclusion criteria

1. Age:18-60 years.

2. White blood cells > 4000/mm^3^

3. Neutrophils > 2000/mm^3^.

4. Platelets > 85000/mm^3^.

5. Prothrombin time < 2 seconds above upper limit of normal (ULN).

6. Direct bilirubin 0.3 mg/dl.

7. Indirect bilirubin 0.8 mg/dl.

8. Albumin > 3.5 gm/dl.

9. Serum creatinine, Fasting blood sugar, TSH within normal limits.

10. HBsAg: Negative.

11. ANA < 1:160.

12. Positive for anti-HCV and HCV RNA.

13. If diabetic, HB A1C < 8.5%.

14. Alpha feto-protein <100 IU/ml., but If is >100, C.T. is normal.

15. Females practice adequate contraception.

16. Male patient's wife practicing adequate contraception.

17. Signed written informed consent for the study.

### Exclusion criteria

Exclusion of,

1. Any other cause of chronic liver disease other than HCV (by liver biopsy).

2. Overt HBV Co-infection

3. Autoimmune disease (by ANA).

4. Alcoholic liver disease.

5. Decompensated liver disease.

6. Hypersensitivity to Peg-IFN/RBV.

7. Pregnancy or breast feeding.

8. Poorly controlled diabetes.

9. Clinically significant retinal abnormalities.

10. Obesity -induced liver disease.

11. Drug-induced liver disease.

12. CNS trauma or active seizures which requires medication

13. Ischemic cardiovascular insult within the last 6 months.

14. Immunologically mediated diseases.

15. Patients with organ transplant.

16. Substance abuse (abstention for the last 12 months).

17. Immunosuppressive drugs.

18. Severe pre-existing psychiatric conditions.

### Patients

In this cross sectional study, patients were divided into two groups according to their HBV DNA status:

**Group I**: Patients having HBV DNA positivity (dually infected patients).

**Group II**: Patients negative for HBV DNA (moninfected patients).

All the studied patients were subjected to the following:

#### I-Clinical assessment

History taking, BMI, physical examination.

#### II. Biochemical assessment

Liver function tests, kidney functions, complete blood counts, serum uric acid, cholesterol and triglycerides, HCV RNA both pre-enrollment and during treatment were examined.

#### III. Liver biopsy

Pathological examination performed at liver histopathology laboratory, Faculty of Medicine, Zagazig University. More than one pathologist revised the slides to minimize inter-observer variability that commonly affects explanation of liver biopsy. Liver biopsies were paraffin-embedded and stained with haematoxylin-eosin and Masson trichrome stains, additional stains were used when needed. The biopsies were reviewed blindly without knowledge of any parameter. Hepatitis grading and staging were evaluated according to the METAVIR scoring system [17].

#### IV. Serodiagnosis of HBV

anti-HBc total antibodies were detected by a rapid immunoassay **(Gold Colloidal Conjugate Membrane, Cal-Tech Diagnostics, INC.- California, USA) **while anti-HBs antibodies were determined by the electro-chemi-luminescence **i**mmuno**a**ssay "ECLIA" technique using commercially available kits **(Cobas e 411 analyzer, Hitachi, Japan)**.

#### V. HBV DNA examination

The assay (done while the patients were under Peg-IFN/RBV therapy) and results were performed and interpreted by investigators who were blinded to the patients' baseline characteristics, HBV serology and stage of liver biopsy. We used COBAS^® ^AmpliPrep/COBAS^® ^TaqMan^® ^HBV Test **(Roche Diagnostics, Switzerland)**, which is a real time quantitative PCR technology. Specimen processing carried out by COBAS AmpliPrep analyzer, specimens were collected by professional staff with sticking to quality standards to avoid any potential contamination, blood was collected in EDTA containing tubes, transfer of the specimen within the laboratory was maintained at 2-8°C, most samples were analyzed on the same day, while when stored for no more than 3 days, they were kept at room temperature (25-30°C). The concentration of HBV DNA in EDTA-plasma that can be detected with a positivity rate of greater than 95% is 12 IU/mL or lower while its specificity is 100%.

### Treatment regimens and follow up

The dose of peginterferon is given subcutaneously around the umbilicus once per week, the dose of alpha-2a is 180 ug together with ribavirin, using 1000 mg/day for those ≤75 kg in weight and 1200 mg/day for those >75 kg in weight; while the dose of alph-2b is 1.5 ug/kg body weight together with ribavirin at dose of 800 mg/day for those weighting <65 kg; 1000 mg for those >65 kg to 85 kg; 1200 mg for those >85 kg to 105 kg; and 1400 mg for those > 105 kg.

### Patient Monitoring

All the patients attended to the Viral Hepatitis out-patient Clinic for monitoring during treatment. Patients were assessed at weeks 0, 1, 2 and 4 of treatment and thereafter monthly. At each review, laboratory tests were performed including serum ALT and AST, bilirubin, full blood count, and serum creatinine. Body weight and symptom checklist were recorded at each visit and dose modifications to the Peg-IFN or ribavirin were made when appropriate. Quantitative serum HCV-RNA was determined at baseline and at week 12. Qualitative HCV-RNA was determined at weeks 24 and 48.

### Statistical analysis

Data were checked, entered and analyzed using SPSS version 13 for data processing and statistics.

## Results

The base line characters of all patients are described in (table 1). Out of the 155 examined chronic HCV patients, only 6 patients (3.9%) had OBI. Comparison between OBI/HCV dually infected and HCV monoinfected patients showed that, dually infected patients are younger in age 32.8 years (p = 0.013), had less advanced hepatic fibrosis (p = 0.02), and higher base line HCV viral load (p = 0.006) than monoinfected patients. Whereas, sex, BMI, liver enzymes, histological activity, HBV serological markers and response rates to Peg-IFN/RBV were comparable between the two groups (table 2).

**Table 1:** Characters of the studied patients.

**Number**	155	
**Age**(years)		
X^- ^± SD	41.8 ± 9.2	
Range	19-59	
**Sex**		
M	125	80.6%
F	30	19.4%
**BMI**		
X^- ^± SD	27.3 ± 3.6	
Range	21- 40.5	
**Duration of HCV infection **(years)		
X^- ^± SD	4.4 ± 3.6	
Range	1-19	
**Smoking status**		
Non	81	52.2%
Active	57	36.8%
Ex-smoker	17	11.0%
**Basal ALT **(× ULN)		
≤2	116	74.8%
> 2	39	25.2%
**Basal AST **(× ULN)		
≤ 2	124	80%
> 2	31	20%
**Activity**		
1	52	33.6%
2	71	45.8%
3	32	20.6%
**Fibrosis**		
1	54	34.8%
2	61	39.4%
3	36	23.2%
4	4	2.6%
**Basal HCV Viral Load **(IU × 10^5^)		
X^- ^± SD	3.87 ± 5.7	
Range	0.001-22.3	

**Table 2:** Biochemical, pathological and virological parameters in HBV/HCV dually infected and HCV mono-infected patients

	Occult HBV/HCV Dual Infection (n = 6)		HCV Monoinfection (n = 149)		Test	P
**Age **(years)					**t Test**	
X^- ^± SD	32.8 ± 6.3		42.2 ± 9.1		2.48	0.013*
Range	20 - 36		19 - 59			
**Sex**	**No**.	**%**	**No**.	**%**	**X^2^**	
M	6	100.0	119	79.9	0.49	0.48
F	0	0.0	30	20.1		
**BMI**					**t Test**	
X^- ^± SD	25.7 ± 1.5		27.3 ± 3.6		1.1	0.27
Range	24 - 27.5		21 - 40.5			
**Basal ALT **(× ULN)	**No**.	**%**	**No**.	**%**	**X^2^**	
≤2	6	100.0	112	75.2	0.83	0.36
> 2	0	0.0	37	24.8		
**Basal AST **(× ULN)	**No**.	**%**	**No**.	**%**	**X^2^**	
≤2	4	66.7	122	81.9	0.16	0.68
> 2	2	33.3	27	18.1		
**Activity**	**No**.	**%**	**No**.	**%**	**X^2^**	
1	2	33.3	50	33.6	1.88	0.39
2	4	66.7	67	44.9		
3	0	0.0	32	21.5		
**Fibrosis**	**No**.	**%**	**No**.	**%**	**X^2^**	
1	0	0.0	54	36.2		
2	6	100.0	55	36.9	9.62	0.02*
3	0	0.0	36	24.2		
4	0	0.0	4	2.7		
**Basal HCV Viral Load **(IU × 10^5^)					**t Test**	
X^- ^± SD	10.05 ± 9.93		3.62 ± 5.4		2.74	0.006*
Range	0.4 - 22.3		0.001 - 22			
**Anti-HBc**	**No**.	**%**	**No**.	**%**	**X^2^**	
Negative	4	66.7	133	89.3	1.09	0.29
Positive	2	33.3	16	10.7		
**Anti-HBs**	**No**.	**%**	**No**.	**%**	**X^2^**	
Negative	6	100.0	131	87.9	0.07	0.79
Positive	0	0.0	18	12.1		
**Response Rate**	**No**.	**%**	**No**.	**%**	**X^2^**	
Responder	0.0	0.0	70	46.98	3.42	0.06
Non-responder	6.0	100	79	53.02		

* Significant

Anti-HBc was associated with higher base line HCV viral load 9.43 × 10^5 ^IU/ml (p < 0.001) and poor response to the combination therapy (p = 0.018), while no relation to histological indices, liver enzymes, HBV DNA nor to Anti-HBs antibodies (table 3).

**Table 3:** Anti-HBc status among studied patients.

	Anti HBc				Test	P
			
	Positive (n = 18)		Negative (n = 137)			
**Age **(years)					**t Test**	
X^- ^± SD	43.4 ± 9.1		41.6 ± 9.2		0.79	0.43
Range	28-59		19-59			
**Sex**	**No**.	**%**	**No**.	**%**	**X^2^**	
M	16	88.9	109	79.6	0.39	0.53
F	2	11.1	28	20.4		
**Activity**	**No**.	**%**	**No**.	**%**	**X^2^**	
1	2	22.15	50	36.5		
2	12	66.7	59	43.1	5.02	0.08
3	4	22.15	28	20.4		
**Fibrosis**	**No**.	**%**	**No**.	**%**	**X^2^**	
1	4	22.2	49	35.7		
2	6	33.3	56	41.0	5.55	0.13
3	8	44.4	28	20.4		
4	0	0.0	4	2.9		
**Basal HCV Viral Load **(IU × 10^5^)					**t Test**	
X^- ^± SD	9.43 ± 9.9		3.1 ± 4.5		4.66	< 0.001*
Range × 10^5^	0.62-22.3		0.001-21			
**Basal ALT**(× ULN)	**No**.	**%**	**No**.	**%**	**X^2^**	
≤ 2	14	77.8	102	74.5	0.001	0.98
> 2	4	22.2	35	25.5		
**Basal AST **(× ULN)	**No**.	**%**	**No**.	**%**	**X^2^**	
≤ 2	16	88.9	108	78.8	0.48	0.49
> 2	2	11.1	29	21.2		
**HBV DNA**	**No**.	**%**	**No**.	**%**	**X^2^**	
Positive	2	11.1	4	2.9	1.09	0.29
Negative	16	88.9	133	97.1		
**Response Rate**	**No**.	**%**	**No**.	**%**	**X^2^**	
Responder	4	22.2	66	48.2	5.58	0.018*
Non-responder	14	77.8	71	51.8		
**Anti-HBs**	**No**.	**%**	**No**.	**%**	**X^2^**	
Positive	4	22.2	14	10.2	1.22	0.26
Negative	14	77.8	123	89.8		

* Significant

Anti-HBs was not associated with any demographic, biochemical, histological, serological parameter abnormality nor to the response rates to combination therapy between positive and negative patients (table 4).

**Table 4:** Anti-HBs status among studied patients.

	Anti HBs				Test	P
			
	Positive (n = 18)		Negative (n = 137)			
**Age **(years)					**t Test**	
X^- ^± SD	43.4 ± 6.5		41.6 ± 9.5		0.79	0.43
Range	29-53		19-59			
**Sex**	**No**.	**%**	**No**.	**%**	**X^2^**	
M	16	88.9	109	79.6	0.39	0.53
F	2	11.1	28	20.4		
**Activity**	**No**.	**%**	**No**.	**%**	**X^2^**	
1	7	38.9	45	32.8		
2	11	61.1	60	43.8	5.42	0.06
3	0	0.0	32	23.4		
**Fibrosis**	**No**.	**%**	**No**.	**%**	**X^2^**	
1	4	22.2	50	36.5		
2	12	66.7	49	35.8	6.6	0.08
3	2	11.1	34	24.8		
4	0	0.0	4	2.9		
**Basal HCV Viral Load **(IU × 10^5^)					**t Test**	
X^- ^± SD	2.3 ± 3.9		4.1 ± 5.9		1.24	0.21
Range	0.006-12.9		0.001-22.3			
**Basal ALT **(× ULN)	**No**.	**%**	**No**.	**%**	**X^2^**	
≤ 2	16	88.9	102	74.5	1.12	0.29
> 2	2	11.1	35	25.5		
**Basal AST **(× ULN)	**No**.	**%**	**No**.	**%**	**X^2^**	
≤ 2	18	100	108	78.8	4.69	0.03
> 2	0.0	0.0	29	21.2		
**HBV DNA**	**No**.	**%**	**No**.	**%**	**X^2^**	
Positive	0	0.0	6	4.4	0.82	0.36
Negative	18	100	131	95.6		
**Response Rate**	**No**.	**%**	**No**.	**%**	**X^2^**	
Responder	10	55.6	60	43.8	0.89	0.34
Non-responder	8	44.4	77	56.2		
**Anti-HBc**	**No**.	**%**	**No**.	**%**	**X^2^**	
Positive	4	22.2	14	10.2	1.22	0.26
Negative	14	77.8	123	89.8		

All dually infected patients were males <40 years of age, had F2 hepatic fibrosis, A1-2 histological activity, all were non-responders to combination therapy, and 2 patients had HCV viral load at time of examination higher than the base line levels (table 5).

**Table 5:** Characteristics of the six HBV/HCV dually infected patients.

Patient	1	2	3	4	5	6
**Age **(years)	20	36	21	37	35	34
**Sex**	M	M	M	M	M	M
**Baseline ALT **(× ULN)	1.5	1	1.5	1	1	1
**Duration of HCV Infection **(years)	1	13	2	12	2	2
**PostTreatment ALT **(× ULN)	1	1	1	1	0.5	0.5
**COBAS HBV DNA Level **(IU/ml)	2980	2210	2880	2010	128	149
**Serological Status**						
**Anti-HBc**	-ve	+ve	-ve	+ve	-ve	-ve
**Anti-HBs**	-ve	-ve	-ve	-ve	-ve	-ve
**METAVIR Score**						
**Activity (A)**	A2	A2	A2	A2	A1	A1
**Fibrosis (F)**	F2	F2	F2	F2	F2	F2
**Basal HCV Load **(IU/ml)	0.4 × 10^5^	22.3 × 10^5^	0.39 × 10^5^	22.1 × 10^5^	7.2 × 10^5^	7.23 × 10^5^
**Post-Treatment HCV Load **(IU/ml)	1.4 × 10^5^	6 × 10^5^	1.38 × 10^5^	5.59 × 10^5^	0.0028 × 10^5^	0.027 × 10^5^
**Response to IFN**	NR	NR	NR	NR	RR	RR

M, male, F, female. -ve, negative, +ve, positive, NR, non responder, RR, relapse

## Discussion

The incidence of OBI in HCV patients varies greatly, ranging from 0%-52% [9,11], in this study the incidence of OBI is 3.9% (only 6 cases of detectable serum HBV DNA with mean HBV DNA level of 1726.2 IU/ml) this low prevalence is in agreement with the rates reported by many investigators [18-20].

Our study, like many other studies, examined only one serum sample, which may not be sufficient to detect OBI if the viral replication is intermittent, and this together with the assumption that OBI is sensitive to Peg-IFN therapy and the intermediate prevalence of HBV in Egypt, 2-8% HBsAg carrier rate [21], may account for the low prevalence of OBI in this study. OBI is characterized by low serum HBV DNA, usually < 200 IU/ml and recent definitions of OBI included liver HBV DNA positivity as a prerequisite for considering OBI [16], but examination of liver tissue is not always applicable in clinical practice [20,22], and that is why highly sensitive PCR assays with low detection limit should be used in diagnosis of OBI [23]. In this aspect Levast et al., (2010) [22] reported liver tissue HBV DNA in 5 out of 113 (4.4%) chronic HCV and none had serum HBV DNA. If occurrence of OBI is thought to be due to strong inhibition HBV replication by the immune system, it thus would be expected to find low levels of HBV DNA in positive cases and this may explain their results [22].

There is a reciprocal inhibition of replication between both HBV and HCV, with dominance of HCV inhibitory effect on HBV replication by its core protein [24], therefore HBV may flare up when the HCV virus is treated [25], and this may explain the slightly high levels of HBV DNA (> 2000 IU/ml) reported in 4 cases of our study, although this is a possible explanation, yet it is not conclusive because we evaluated HBV DNA at only one point of time. If occurrence of OBI would be due to HBV surface mutants, that test negative for HBsAg by the commonly available commercial kits [26], and that may have levels of viraemia comparable to overt infection [27], it may be another explanation for the high serum HBV DNA levels in our study. Long lasting persistence of HBV in the liver may provoke a mild but continuing necroinflammation, that, if other causes of liver damage (such as HCV) co-exist, may over time contribute to progression of chronic liver disease to cirrhosis [8,28], this is shown in our patients where all dually infected patients were F2 and 4 cases were A2.

In this study OBI could not be predicted by serological markers of HBV infection, where only 2 out of the 6 patients with detectable HBV DNA had anti-HBc antibodies, and none had anti-HBs antibodies. Anti-HBc antibody was present in 18 patients with incidence of 11.6%. Anti-HBc was associated with anti-HBs in 4 patients (2.6%), while anti-HBc only state was reported in 14 patients (9%). This can be explained by the notion that following HBV infection both anti-HBs and anti-HBc antibodies develop, while titres of anti-HBs decreases over years to undetectable levels, anti-HBc antibodies only persists [29].

We reported non significant differences in histological activity and fibrosis between anti-HBc positive and negative patients and also between anti-HBc positive/DNA positive and anti-HBc positive/DNA negative patients, these results disagree with Yuki et al. (2003) [30] and El-Sharaway et al. (2007) [31]. Our results are in agreement with those of Chen et al. (2010) [19]and Levast et al. (2010) [20], who reported lower and similar rates of histological activity and hepatic fibrosis between OBI/HCV patients and HCV monoinfected patients respectively. These results seems not to be related to HCV genotypes, where our patients are mostly of genotype 4, that is known in Egypt to be extremely variable, not only in terms of sequence, but also in terms of functional and immunological determinants [32] in comparison to non-genotype 4 in their studies.

Anti-HBc is associated with higher basal HCV viral load and non-response to Peg-IFN/RBV therapy, but these findings needs to be confirmed in further studies, especially with the trend in the literature to neglect seromarkers of HBV in defining OBI.

Anti-HBs was not linked to non-response to IFN therapy in previous studies; this is also applied to our study, where no statistically significant difference as regard response rates to Peg-IFN based therapy was noticed. But, it should be evident that anti-HBs positive cases showed no correlation to advanced necroinflammation and fibrosis in our study, in contrast to what had been noticed by some authors [33].

As regard to impact of OBI on the response to standard interferon/ribavirin therapy, authers found a non significant results between patients with and without OBI [13,34-36]. In contrast low response rates were recorded in chronic HCV patients with OBI under standard interferon monotherapy [6,9-12]. Our study uses the standard of care Peg-IFN/RBV therapy, that potentially cures OBI. Levast et al., (2010) [20], conducted retrospective analysis of 140 patients treated for chronic HCV by Peg-IFN/RBV. They confirmed the lack of association between HBV markers (HBV DNA, anti-HBc, and anti-HBs antibodies) and disease severity or response to treatment, we reported similar findings for HBV DNA and anti-HBs while anti-HBc was linked to non-response to the combination therapy.

Chen et al., (2010) [19] concluded that, OBI was rare in HCV, virological and biochemical features between OBI/HCV patients and HCV monoinfection patients were comparable, these findings are similar to our findings, while HBV DNA in dually infected patients was low and patients with OBI responded to Peg-IFN/RBV therapy, in contrast to high HBV DNA load in our patients and lack of response in our 6 patients, this may be related to HBV genotypes, where HBV genotype D is the prevalent genotype in Egypt.

Patients with HCV/HBV dual infection were noticed to have high HCV RNA load than those with HCV mono-infection [11,37]. This seems to be applicable to genotype 4, where HBV DNA positive patients in our study showed higher baseline HCV viral load than HCV monoinfected patients. Suppression of the dominant virus -usually HCV predominates over HBV- may be associated with flares of the non-dominant virus, and this notion is reflected by the relatively high levels of HBV DNA in our positive cases (1726.1 IU/ml), in comparison to 200 IU/ml proposed by Raimondo et al. [16] and 923 IU/ml reported by Chen et al. [19], this may be related to viral genotypes where our cases are HCV genotype 4 and HBV genotype D whose clinical impact has been studied less extensively [38].

HBV DNA positive cases in our study tends to be of younger age group (32.8 ± 6.3 years), this may be due to more risk of exposure to infection

## Conclusions

In conclusion, detection of HBV DNA in HBsAg negative Egyptian chronic HCV patients is not a statistically significant cause of non-response of those patients to the current standard of care Peg-IFN/ribavirin therapy, and hence relying on the available data it is not currently recommended to screen for OBI in chronic HCV before initiation of antiviral therapy. The finding that anti-HBc antibody may be associated with non-response to antiviral therapy needs further confirmation. Anti-HBs seems to have no relation with any biochemical, pathological nor virological aspects in these patients. Dually infected patients are of younger age group, had higher baseline HCV viral load and were non responders to antiviral therapy.

## List of abbreviations

A: Activity; BMI: Body Mass Index; F: Fibrosis; HBV: Hepatitis B Virus; HCC: Hepatocellular Carcinoma; HCV: Hepatitis C Virus; IU: International Unit; NR: Non-Responder; OBI: Occult Hepatitis B Infection; PEG-IFN: Pegylated Interferon; RBV: Ribavirin; RR: Relapser; SVR: Sustained Virological Response; ULN: Upper Limit of Normal.

## Competing interests

The authors declare that they have no competing interests.

## Authors' contributions

MHE: participated in the study design planning, collection of cases, obtaining institutional and patients consent, and writing the manuscript, NEEG: participated in the study design planning, collection of cases, summarization of data and statistical analysis, LAM: participated in the study design planning, laboratory analyses including serology and DNA, and statistical analysis, MMB: participated in the study design planning, revision of patients' original records, obtaining institutional and patients consent, and writing the manuscript. All authors read, revised and approved this final manuscript.
